# Uncommon Cause of Trigeminal Neuralgia: Tentorial Ossification over Trigeminal Notch

**DOI:** 10.1155/2015/819354

**Published:** 2015-08-24

**Authors:** Sun Woo Bang, Kyung Ream Han, Seung Ho Kim, Won Ho Jeong, Eun Jin Kim, Jin Wook Choi, Chan Kim

**Affiliations:** ^1^Department of Radiology, Kim Chan Hospital, 228 Hyowon-ro, Gwonseon-gu, Suwon 441-822, Republic of Korea; ^2^Department of Pain Medicine, Kim Chan Hospital, 228 Hyowon-ro, Gwonseon-gu, Suwon 441-822, Republic of Korea; ^3^Department of Cardiovascular Surgery, Kim Chan Hospital, 228 Hyowon-ro, Gwonseon-gu, Suwon 441-822, Republic of Korea

## Abstract

Ossification of the tentorium cerebelli over the trigeminal notch is rare, but it may cause compression of the trigeminal nerve, leading to trigeminal neuralgia (TN). We were unable to find any previously reported cases with radiological evaluation, although we did find one case with surgically proven ossification of the tentorium cerebelli. Here, we present a case of TN caused by tentorial ossification over the trigeminal notch depicted on magnetic resonance imaging (MRI) and computed tomography (CT).

## 1. Introduction

Calcifications or ossifications in the free edges of the tentorium cerebelli, in the so- called tentorial notch, are frequent and age-related physiologic and neurodegenerative changes [1]. However, ossification of the tentorium cerebelli over the trigeminal notch is rare and may cause compression of the trigeminal nerve, leading to trigeminal neuralgia (TN) [2]. We report a case of TN secondary to tentorial ossification over the trigeminal notch depicted on magnetic resonance imaging (MRI) and computed tomography (CT).

## 2. Case Description

**Figure 1:**
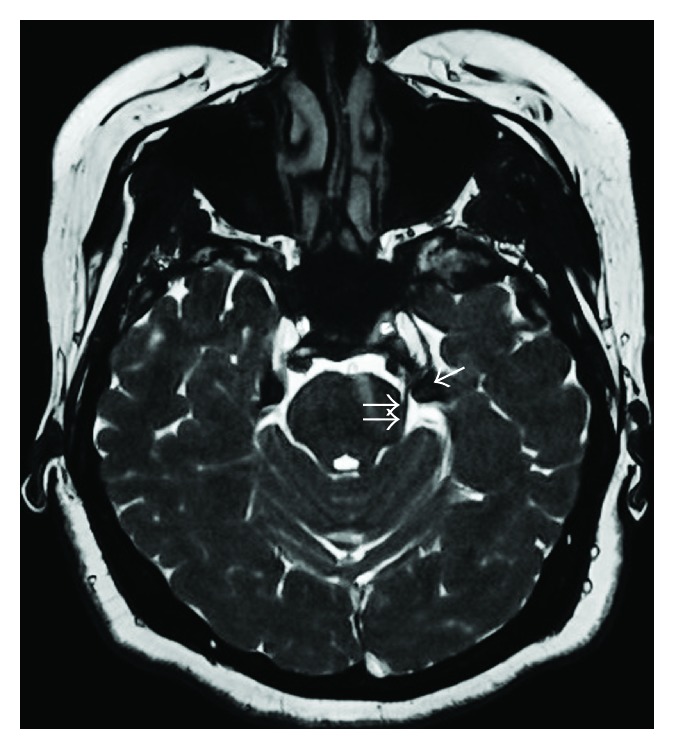
Axial 3D FIESTA MR image shows nodular lesions (arrow) with dark signal intensity adjacent to the porus trigeminus bilaterally. The cisternal segment of the left trigeminal nerve (double arrows) is impinged by nodular lesions.

**Figure 2:**
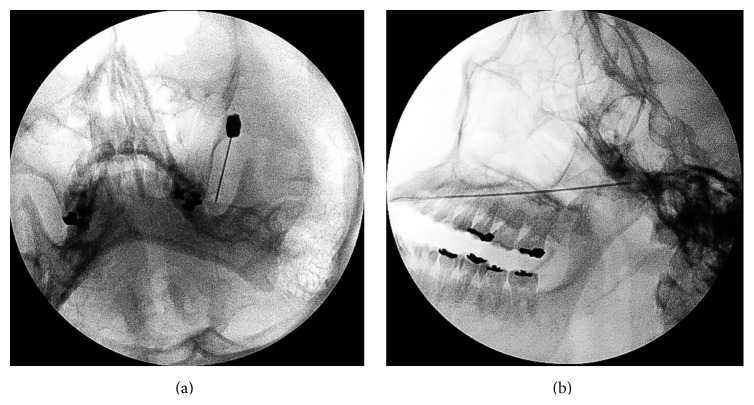
Fluoroscopy-guided diagnostic block of the left mandibular nerve. Anteroposterior (a) and lateral (b) views show the needle tip directed toward the left foramen ovale.

**Figure 3:**
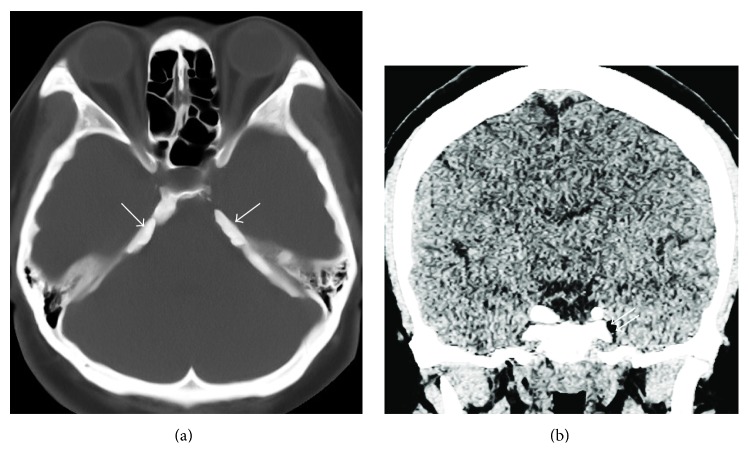
Brain CT image shows plaque-like tentorial ossification (arrow) on bone window (a) and an air bubble (double arrows) adjacent to left Meckel's cave on brain window (b).

A 35-year-old woman visited the hospital with a chief complaint of left facial pain that had lasted one week. She had experienced three episodes of the pain over the past four years. Each pain attack lasted three or four months, with a pain-free period. The pain affected the left maxillary and temporal regions and the inside of the ear. The nature of the pain was electric-shock-like, paroxysmal, and lancinating. The intensity of the pain was 10 on the visual analogue scale (no pain is 0; imaginary maximal pain is 10). The pain was aggravated or precipitated by talking, swallowing, opening the mouth, and eating. Neurologic examination was negative including sensory deficits. The clinical diagnosis was TN in the left maxillary and mandibular distribution. A brain MRI was performed to rule out other causes of the patient's facial pain. The MRI scan was obtained using a 1.5 Tesla MRI scanner (Signa HDxt, GE Healthcare Systems, Wauwatosa, WI, USA). Axial MR images with 3D fast imaging with steady-state acquisition (3D FIESTA) sequence (TR, 5.1 ms; TE, 2.0 ms; slice thickness, 1.0 mm; FOV, 22 × 22 cm; matrix, 320 × 320; and NEX, 1) showed nodular lesions with dark signal intensity adjacent to the porus trigeminus. The lesions were impinging upon the cisternal segment of the left trigeminal nerve (Figure 1). There was no evidence of vascular contact or compression of the cisternal segment of the left trigeminal nerve. The radiological diagnosis was ossification of the tentorium cerebelli over the trigeminal notch. A pain intervention doctor performed a diagnostic block of the left mandibular nerve with local anesthetics (Figure 2) and the patient's pain disappeared for about one hour. A brain CT (Brivo CT385, GE Healthcare Systems) obtained after the diagnostic block showed dense plaque-like ossification of the tentorium cerebelli over the trigeminal notch and an air bubble adjacent to left Meckel's cave (Figure 3). The patient was unable to eat meals or talk even after medications. Informed consent was obtained for a left mandibular nerve block with alcohol. Under fluoroscopic guidance, a left mandibular nerve block was performed with 0.5 mL of alcohol, and the patient's pain was completely relieved.

## 3. Discussion

The tentorium cerebelli is one of the most common sites of intracranial physiologic calcification [1]. In fact, the mineralization of the tentorium as visualized on radiographs is ossification rather than calcification [2]. Intracranial physiologic ossifications are generally unaccompanied by any evidence of disease and have no demonstrable pathologic cause. Ossifications of the falx, dura mater, or tentorium cerebelli occur in about 10% of the elderly population. Dural and tentorial ossifications usually have a laminar pattern and can occur anywhere within the cranium [1]. However, ossification of the tentorium cerebelli over the trigeminal notch is rare and is a possible cause of TN [2]. Anatomically, the porus trigeminus, the opening of Meckel's cave, is bounded superiorly by the tentorium cerebelli and inferiorly by the trigeminal notch [2]. Standefer et al. reported a clinical case of TN secondary to tentorial ossification. However, the site of ossification was near the tentorial notch, somewhat apart from the trigeminal notch. Furthermore, there was no description of radiological evaluation such as by MRI and CT of the tentorial ossification; there was merely an artist's depiction of the operative procedure [3]. According to the International Classification of Headache Disorders, the etiology of TN is divided into classical TN, caused by vascular compression of the trigeminal root entry zone, and symptomatic TN, caused by tumors, demyelination, and vascular disorders [4]. Routine brain MRI reveals structural causes in up to 15% of TN patients [5]. The most common causes of symptomatic TN are cerebellopontine angle tumors and multiple sclerosis. As we know, typical ossification of the tentorium cerebelli shows laminar or mildly nodular patterns and is a clinically insignificant finding [1]. But if the ossification is prominent, as observed in our case, compression of the cisternal segment of the trigeminal nerve is possible and symptomatic TN may develop.

On initial examination, the patient in this case presented with paroxysmal pain in the temporal area and inner ear triggered by swallowing and the authors considered that the most likely diagnosis would be trigeminal neuralgia combined with glossopharyngeal neuralgia. However, diagnostic mandibular nerve block produced complete pain relief for the duration of action of the local anesthetic, and a subsequently performed neurolytic mandibular nerve block led the patient to be pain-free.

We have described a case of TN caused by unusually prominent ossification of the tentorium cerebelli over the trigeminal notch that was depicted on MRI and CT.
